# Ossifying renal tumor of infancy: A case report

**DOI:** 10.1016/j.eucr.2024.102686

**Published:** 2024-02-15

**Authors:** Saad Andaloussi, Omar Dalero, Aziz Elmadi

**Affiliations:** a Department of Pediatric Surgery, University Hospital Mohamed VI, Tangier, Morocco; b Faculty of Medicine and Pharmacy of Tangier, Abdelmalek Essaâdi University, Tangier, Morocco

**Keywords:** Ossifying renal tumor of infancy (ORTI), Renal tumor, Hematuria, Infant

## Abstract

Ossifying Renal Tumor of Infancy (ORTI) represents an extremely rare and benign renal neoplasm, with limited cases published in the literature. Predominantly characterized by painless and intermittent gross hematuria, the diagnostic evaluation is effectively facilitated through ultrasound, computed tomography, and magnetic resonance imaging. Despite progress, its etiology has not yet been elucidated. We report an additional case with an unusual clinical presentation.

## 1 Introduction

Ossifying Renal Tumor of Infancy (ORTI) is an extremely rare benign renal tumor with limited cases documented to date. It was initially described by Chatten et al., in 1980. Typically affecting male infants, ORTI manifests with intermittent episodes of painless gross hematuria. Occasionally, an abdominal mass may be present; however, arterial hypertension has not been reported in the literature. The radiologic appearance of ORTI may potentially lead to confusion with renal calculus disease or a calcified Wilms tumor. Recognition of ORTI is crucial because neither chemotherapy nor radiotherapy is deemed necessary for its management. Surgical excision is curative, and recurrence or malignant transformation have not been documented.

## 2 Case presentation

**Fig. 1:**
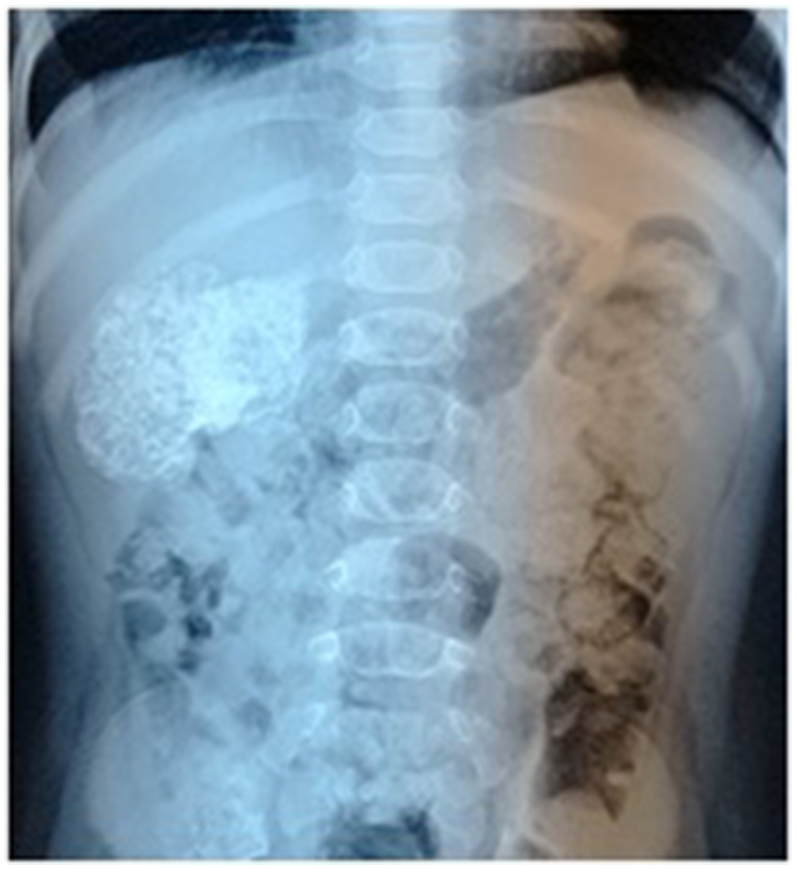
Plain radiograph shows flecks of calcifications in the expected area of the right kidney.

**Fig. 2:**
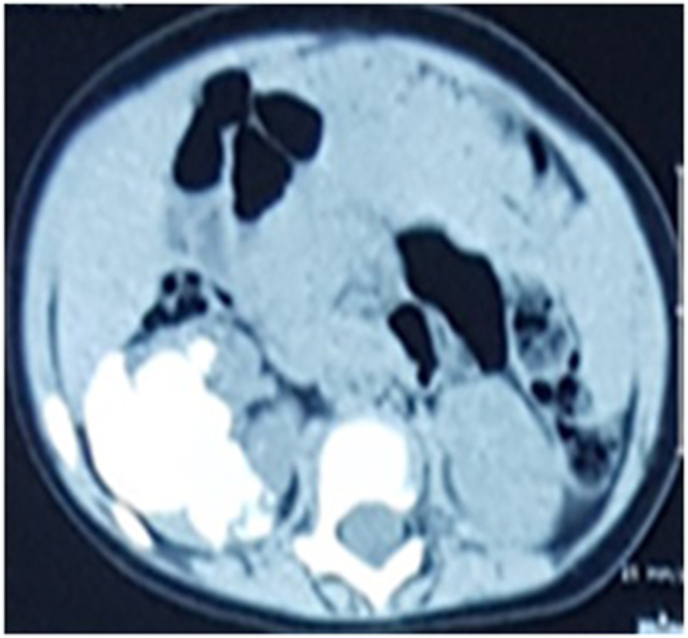
Axial nonenhanced CT image shows a calcified renal mass located in the upper pole and central part of the right kidney with no tissue portion visible.

**Fig. 3:**
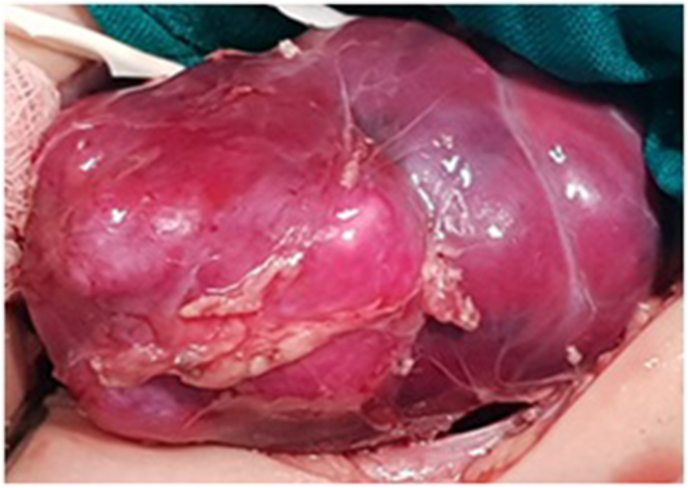
Resected specimen revealed an ossifying-like appearance that occupied the right kidney.

We describe a case of an 8-month-old boy who referred to our hospital with painless intermittent gross hematuria since the age of 2 months. The parents denied any history of fever or trauma. The child didn't show any other clinical symptoms and no associated anomalies were seen. He had no signs of a urinary tract infection. The physical examination was unremarkable except for arterial hypertension; the blood pressure was 128/70. The patient's abdomen was soft, not distended, without a palpable mass or tenderness to palpation. Urinalysis results showed numerous red blood cells per high-power field. Neither bacteria nor nitrites were found. Renal function and electrolyte examinations returned normal results. Plain radiograph revealed flecks of calcifications in the expected area of the right kidney (Fig. 1). Ultrasonography examination showed a solid calcified mass in the upper pole and central part of the right kidney with no distortion of the renal contour. No obvious hydronephrosis or ureter dilatation was seen and no abnormalities were identified in the other kidney. Non-contrast computed tomography revealed a 45 mm benign looking renal mass, calcified, located in the upper pole and central part of the right kidney with no tissue portion visible (Fig. 2). No other associated anomalies were identified. Initially, a partial nephrectomy was considered via an open transabdominal approach; however, the persistence of arterial hypertension, despite the total resection of the tumor, prompted us to complete with a radical nephrectomy of the right kidney. Intraoperatively, no obvious macroscopic extension of the tumor was identified and the procedure was conducted without complication (Fig. 3). The patient had an uncomplicated hospital stay and was discharged on the fifth postoperative day. Pathology analysis subsequently confirmed the diagnosis of ORTI. At his 4-year follow-up, the patient was clinically well without hematuria or arterial hypertension, and repeated abdominal ultrasounds showed no signs of recurrence on the ipsilateral or contralateral sides.

## 3 Discussion

ORTI represents an exceedingly rare and benign renal tumor typically observed in early infancy. It was initially described as a distinct variant of pediatric renal tumors by Chatten in 1980.^1^ To our knowledge, only 30 cases of ORTI have been reported in the literature.^2^ Concerning its pathogenesis, some publications suggest that it may arise from the epithelium of the urinary tract rather than the renal parenchyma, others believe that it is a subtype of congenital mesoblastic nephroma while no cases of associated Wilms tumor have been reported.^3^ Microscopically, the tumor presents three major histologic components: osteoblast-like cells within and at the periphery of the core, spindle cells, and an osteoid core. The proportion of these components varies, but the proportion of osteoid and degree of osseous maturation augment with increasing age of the patient. Compared to the usual renal tumors found in infancy, the histological features of our case are distinctive. This ossifying tumor does not display the triphasic pattern characteristic of a typical nephroblastoma, which consists of stroma, blastema, and epithelium. In addition, clonal trisomy 4 has been identified as a cytogenetic feature of ORTI, distinguishing it from Wilms tumor with calcification.^4^ No case presented malignant transformation or recurrence of the tumor.

Clinically, ORTI generally occurs during early childhood, most often in male patients less than 1 year old. Painless intermittent gross hematuria is the most common symptom. Although less common, an abdominal mass may also be present. No patient presented with arterial hypertension, a distinction observed in our case. Associated hypertension is related to an increase in renin activity caused by secretion from entrapped juxtaglomerular cells. In imaging studies, a plain abdominal radiograph serves to confirm calcification within this region. Ultrasound typically reveals an echogenic mass with shadowing. Computed tomography and magnetic resonance imaging with contrast can be useful in the differential diagnosis of ORTI. Calculi are very rare in infants, even with metabolic disease associated with crystalluria. The primary radiological differential diagnosis is Wilms tumor versus ORTI. It is important to recognize this distinct tumor because no chemotherapy or radiotherapy is necessary. Treatment is based on complete resection of the mass with preservation of the renal parenchyma through an open transabdominal approach. A safe laparoscopic nephrectomy in children with ORTI has been reported.^5^ However, in specific cases, a radical nephrectomy may be considered necessary such as observed in our patient due to the persistence of arterial hypertension. Subsequently, a prolonged follow-up is imperative to ensure the absence of recurrence.

## 4 Conclusion

ORTI is an exceptional renal tumor in children. It must be considered in the differential diagnosis in cases of intermittent episodes of painless macroscopic hematuria occurring in the pediatric population, especially when associated with a non-invasive calcified renal mass on radiology. However, this case report recommends a systematic evaluation of arterial hypertension, potentially influencing the therapeutic approach.

## Funding

This research did not receive any specific grant from funding agencies in the public, commercial, or not-for-profit sectors.

## Informed consent

Patient's attendant (father) provided informed consent prior to study participation.

## Declarations of interest

None.

## CRediT authorship contribution statement

**Saad Andaloussi:** Writing – review & editing, Writing – original draft, Validation, Conceptualization. **Omar Dalero:** Visualization. **Aziz Elmadi:** Validation, Supervision.
